# Dynamics of the seasonal airborne propagation of *Staphylococcus aureus* in academic dental clinics

**DOI:** 10.1590/1678-7757-2017-0141

**Published:** 2018-03-26

**Authors:** Wagner Luiz de Carvalho Bernardo, Jeferson Júnior da Silva, José Francisco Höfling, Edvaldo Antônio Ribeiro Rosa, Marcelo Fabiano Gomes Boriollo

**Affiliations:** 1 Universidade Estadual de Campinas, Faculdade de Odontologia de Piracicaba, Departamento de Diagnóstico Oral, Laboratório de Microbiologia e Imunologia, Piracicaba, São Paulo, Brasil; 2 Universidade José do Rosário Vellano, Faculdade de Medicina, Laboratório de Farmacogenética e Biologia Molecular, Alfenas, Minas Gerais, Brasil; 3 Pontifícia Universidade Católica do Paraná, Escola de Ciências da Vida, Unidade de Pesquisa com Xenobióticos, Curitiba, Paraná, Brasil

**Keywords:** Staphylococcus aureus, Dentistry, Environment, Molecular biology, Genetic diversity

## Abstract

**Objective:**

*Staphylococcus aureus* strains can be disseminated during dental treatments and occasionally lead to the contamination and infection of patients and dentists, which is an important public health problem. The dynamics of the airborne propagation and the genetic diversity of *S. aureus* isolated in an academic dental clinic environment were investigated using isoenzyme typing.

**Material and Methods:**

The isoenzymes of 44 previously reported isolates were obtained from fresh cultures and extracted using glass beads. Nine isoenzymes were investigated using multilocus enzyme electrophoresis (MLEE). The genetic diversity and relationship among the strains (electrophoretic type – ET) were determined using statistics previously described by Nei^25^ (1972) and the SAHN grouping method (UPGMA algorithm).

**Results:**

Clonal pattern analyses indicated a high level of genetic polymorphism occurring among the 33 ETs, which were grouped into five taxa. Each taxon presented one or more clusters that were moderately related and that contained two or more identical/highly related isolates, revealing seasonal airborne propagation in these dental clinic environments.

**Conclusions:**

These data suggest the occurrence of active microevolutionary processes in *S. aureus* as well as the possibility of environmental propagation during a 14-month time span. Such findings are important to show that multiuser academic dental clinics can retain certain strains that are spreadable to different niches.

## Introduction

The dissemination of *S. aureus* is an important public health problem because its resistant strains are involved in severe infections, predominantly in children and hospitalized patients^21^. Dental practitioners treat a wide range of patients. Therefore, it is likely that they will have contact with people colonized or infected with drug-resistant microorganisms^4^. High resistance rates against the antibiotics used for prophylaxis in Dentistry have been detected for pathogens associated with bacterial endocarditis such as *S. aureus* ^19^. *S. aureus* strains can be disseminated during dental treatments and occasionally lead to the contamination and infection of patients and dentists^24^. Certain aspects of practicing Dentistry may contribute to the transmission of microorganisms. Skin, environment, and instruments can be contaminated with saliva, blood, or organic debris during routine dental treatment^29^. Several investigators have observed an increase in the amount of microorganisms during clinical procedures in a dental environment, suggesting contamination from aerosols and especially when high-speed handpieces or ultrasonic scalers are used^1^ ^,^ ^20^. Among the species identified by microbiological studies, *Streptococcus viridans* and *Staphylococcus* spp. are the most prevalent microorganisms found on the surfaces of dental equipment^12^, which includes the methicillin-resistant *S. aureus* that has been detected on the surfaces of dental operatories, air-water syringes, and reclining chairs^18^.

Phenotype- and genotype-based methods have allowed researchers to classify microbial isolates in systematic, taxonomic, evolutionary, phylogenetic, and epidemiological studies^2^ ^,^ ^28^. MLEE has been used for several decades as a standard method in eukaryotic population genetics and systematics^26^ as well as in large-scale studies to estimate the genetic diversity and structure in natural populations of a variety of bacterial^23^, fungal, and yeast^3^ species. Its special advantage is that mobility variants (electromorphs or allozymes) of an enzyme can be directly equated with alleles at the corresponding structural gene loci. Another attractive feature is the likelihood that much of the electrophoretically demonstrable polymorphic variation in enzymes is selectively neutral or nearly so and, therefore, minimally subjected to evolutionary convergence^2^ ^,^ ^28^. The genetic interpretation of patterns depends on the ploidy of the organism, and different rules have to be applied for haploid and diploid organisms^2^ ^,^ ^28^. Based on these rules, the allelic makeup of each isolate is determined over the set of different enzymes studied (between 10 and 30). The enzymes examined in MLEE typically participate in the basic metabolism of the cell and are less likely than other loci to be under selective pressure from the environment or to be subjected to convergence^2^. Therefore, MLEE data are considered representative of the whole genome of an organism and form a good basis for population genetic studies of bacteria, fungi, and protozoa^2^. The genetic data obtained by MLEE can be used for a variety of purposes in the medical microbiology field: (i) to infer the degree of genetic recombination occurring within a natural population, (ii) to assess the degree of genetic isolation of natural populations, either for geographical and ecological reasons or because of biological barriers, (iii) to assess the degree of genetic relatedness between organisms or populations, and thus, provide systematic and taxonomic implications, (iv) to identify bacterial clones within species that can be associated with particular clinical patterns, with a particular frequency in clinical diseases or with a higher level of pathogenicity, and (v) epidemiological tracing of microorganisms. This method represents an invaluable complement to the most recent molecular typing methods, particularly for large-scale epidemiological studies^2^. In addition, MLEE possesses an excellent typeability (i.e., the percentage of distinct strains obtained), excellent reproducibility (i.e., the percentage of strains that show the same results in repeated assays), and good discriminatory power (i.e., its ability to differentiate unrelated strains)^2^ ^,^ ^3^.

Here, we evaluated the genetic diversity and the distribution of airborne *S. aureus* in a multi-user academic clinic by MLEE and a clustering analysis in an attempt to understand the air-spreading behavior of this bacterium.

## Material and methods

### Bacteria

A total of forty-four *S. aureus* isolates (predominantly ampicillin-resistant and β-lactamase positive) were employed. The bacteria were isolated, identified, and had their resistance profiles performed as described in a previous study^1^. Briefly, these isolates were passively harvested from a clinical environment (the air) in the academic clinic (Dental School). Open plates containing MSA (mannitol salt phenol-red agar) selective medium (Merck; Darmstadt, Germany) were placed in 12 dental clinic environments for two hours during intense and periodical multi-activities. This procedure was conducted for 12 months twice a month with intervals of ±15 days between one harvest and another, totaling 24 collections (from September to mid-December, 2000; from March to June, 2001; from August to mid-December, 2001). The plates were then incubated at 37°C for 48 h. Colonies indicating mannitol fermentation by staphylococci were selected, and the characterization of *S. aureus* was performed by Gram staining; growth in a chromogenic medium CHROMagar™ *Staph aureus* (Probac do Brasil Produtos Bacteriológicos Ltda.; São Paulo, SP, Brazil); and catalase, coagulase, and β-lactamase tests using Cefinase™ discs (Becton, Dickinson and Company; Franklin Lakes, NJ, EUA). Antimicrobial susceptibility testing was also performed^1^.

### Enzyme extraction

Bacterial cultures were grown in flasks containing 200 mL BHI (Brain Heart Infusion) culture medium (HiMedia Laboratories GmbH; Einhausen, Germany) at 37°C for 24 h under constant shaking at 150 rpm. After growth, the cells were centrifuged at 5,000×*g* for 3 min and washed in sterile 40 mM PBS (pH 7.5) three times. Pellets (~250 mg) were transferred to 2 mL microtubes containing 250 mL PBS and ~250 mg glass beads (0.45-0.55 mm). These mixtures were kept on ice (4°) for 5 min and then agitated 4 times in a BeadBeater® (Biospec Products, Inc.; Bartlesville, OK, USA) at 4,200 rpm for 30 s with one-minute intervals. Cell fragments were centrifuged at 5,000×*g* for 5 min at 4°C using a centrifuge 5403, rotor 16F24-11 (Eppendorf AG; Hamburg, Germany). The resulting upper aqueous phase was applied to Whatman #3 filter papers wicks (12×5 mm) and maintained at -70°C until use^3^ ^,^ ^28^.

### Electrophoresis and specific enzyme stainings

**Figure 1:**
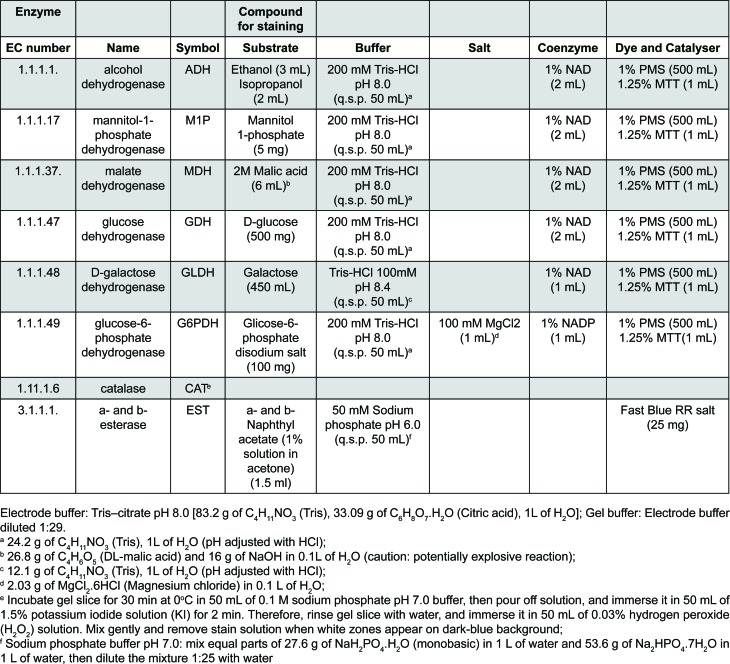
Systems and solutions utilized for the multilocus enzyme electrophoresis (MLEE) analyses of the *S. aureus* metabolic enzymes

The enzymes were separated in 13% (wt/vol) Penetrose 30® starch gels (dimensions 200×120×10 mm) (Refinações de Milho Brasil Ltda.; Mogi-Guaçu, SP, Brazil) in Tris-citrate buffer pH 8.0 (electrode buffer diluted 1:29)^28^. The wicks were then immediately soaked in 5 mL [0.02% (wt/vol)] bromophenol-blue solution and then perpendicularly applied to a longitudinally cut gel (20 mm). Electrophoresis was performed in a horizontal and continuous system at 130 volts at 4°C overnight (bromophenol-blue migration equivalent to 80 mm) using Tris-citrate electrode buffer (pH 8.0). To assure reproducible results, *S. aureus* ATCC® 29213 enzymes were included in each gel. After the electrophoresis, the gels were put on acrylic base and sliced into 1.5-mm sections with the aid of rulers and #15 nylon strings. The sections were carefully placed inside white porcelain containers and stained for 9 enzymes (14 enzymatic loci)^28^. The enzymatic activities that were analyzed included: alcohol dehydrogenase (EC 1.1.1.1), mannitol-1-phosphate dehydrogenase (EC 1.1.1.17), malate dehydrogenase (EC 1.1.1.37), glucose dehydrogenase (EC 1.1.1.47), D-galactose dehydrogenase (EC 1.1.1.48), glucose-6-phosphate dehydrogenase (EC 1.1.1.49), catalase (EC 1.11.1.6), and a- and b-esterase (EC 3.1.1.1.) (Figure 1). The enzymatic expression levels of alcohol dehydrogenase (Adh-1 and Adh-2), mannitol-1-phosphate dehydrogenase (M1p-1 and M1p-2), malate dehydrogenase (Mdh-1 and Mdh-2), and a- and b-esterase (α-Est-1, α-Est-2, β-Est-1 and β-Est-2) identified two genetically interpretative loci.

### Genetic interpretation of the MLEE patterns

Pattern interpretation was performed following the general rules commonly accepted in the deduction of allelic composition for haploid organisms. The bands on the gels were numbered in order of decreasing mobility, and their corresponding alleles were numbered by using the same nomenclature. A lack of demonstrable activity for an enzyme was scored as one null allele at the corresponding gene locus. Each unique combination of alleles over the 14 enzyme loci examined resulted in an electrophoretic type (ET) – subtype or strain^28^.

### Discriminatory power

The discriminatory power of the MLEE method, which is based on the numeric interpretation of electrophoretic patterns, was established using the numerical index of discrimination (*D*), which is according to the probability that two unrelated isolates sampled from the test population will be classified into different types (i.e., strains or ETs). This probability can be calculated using Simpson's index of diversity, developed to describe species' diversity within an ecological habitat. This index may be derived from an elementary probability theory and is given by the following equation: $D=1-\frac{1}{N\left(N-1\right)}\sum n_{j}\left(n_{j}-1\right)$ where *N* is the total number of isolates in the sample population, *S* is the total number of types (strains) described, and *n_j_* is the number of isolates belonging to the *j^th^* type (strain). This equation was derived as follows. The probability that two isolates sampled consecutively will belong to that type (strain) is: $\frac{n_{j}\left(n_{j}-1\right)}{N\left(N-1\right)}$

These probabilities can be summed for all the described types (strains) to determine the probability that any two consecutively sampled isolates will be the same type (strain). This summation can be subtracted from 1 to obtain the equation above. This equation can be applied both to a direct comparison of the discriminating power of typing methods and to an analysis of the discriminating power of the combined typing schemes. To interpret the typing results with confidence, an index greater than 0.90 is desirable^3^.

### Cluster analysis

The statistics of Nei^25^ (1972) were used to estimate the genetic distance among the *S. aureus* isolates: *d_ij_* = - *In*(*I*) or

$$.d_{ij}=-In[\frac{\sum \left|x_{ki}x_{kj}\right|}{\sqrt{\sum x_{ki}^{2}x_{kj}^{2}}}].,$$

where *I* is the normalized identity of genes between two populations (ranging from 0 to infinity), a measure of genetic distance based on the identity of genes (frequency of alleles for all loci, including monomorphic loci) among populations. This genetic distance measures the accumulated allelic differences *per* locus, which can also be estimated from the amino acid sequences of proteins and even for a distantly related species. Therefore, if enough data are available, the genetic distances between any pair of organisms can be measured in terms of *d_ij_* In addition, this measure is applicable to any type of organism regardless of ploidy or matting scheme. Its interpretation in terms of enzyme loci infers that, on average, 0 to an infinite number of allelic substitutions are detected (by electrophoresis) in every 100 loci, from a common ancestral strain^3^ ^,^ ^25^. A tree with two-dimensional classifications (dendrogram), based on matrix *d_ij_*, was generated using the SAHN grouping method (Sequential, Agglomerative, Hierarchic, Nonoverlapping Clustering Methods) and UPGMA algorithm (Unweighted Pair-Group Method using an Arithmetic Average)^3^. Because MLEE provides all levels of relatedness, which must be resolved by DNA fingerprinting methods (i.e., identifying the same strain in independent isolates, identifying microevolutionary changes in a strain, identifying clusters of moderately related isolates, and identifying completely unrelated isolates), a threshold (average value:*d_ij_*) was established in the dendrograms to identify clusters of identical isolates and highly related isolates and *taxa* (singular *taxon*, i.e., a taxonomic group of any nature or rank) (0£*d_ij_*< *d_ij_*)^3^.

The Pearson product–moment correlation coefficient (ranging from -1 to + 1),

$$.r_{jk}=\frac{\sum_{i=1}^{n}\left(X_{ij}-\bar{X_{j}}\right)\left(X_{ik}-\bar{X_{k}}\right)}{\sqrt{\sum_{i=1}^{n}{\left(X_{ij}-\bar{X_{j}}\right)}^{2}\sum_{i=1}^{n}{\left(X_{ik}-\bar{X_{k}}\right)}^{2}}}.,$$

[where *X_ij_* stands for the character state value of character *i* in the operational taxonomy unit (OTU) *j*, *X_i_*is the mean of all state values for OTU *j*, and *n* is the number of characters sampled], was used as a measure of the agreement between the genetic distance values implied by the UPGMA dendrograms and the ones from the original genetic distance matrices (*d_ij_*). These agreements were interpreted as follows: *r*>0.9, very good fit; 0.8≤*r*<0.9, good fit; 0.7≤*r*<0.8, poor fit; *r*<0.7, very poor fit. All of these analyses were obtained using the NTSYS-pc 2.1 program^3^.

## Results

**Figure 2:**
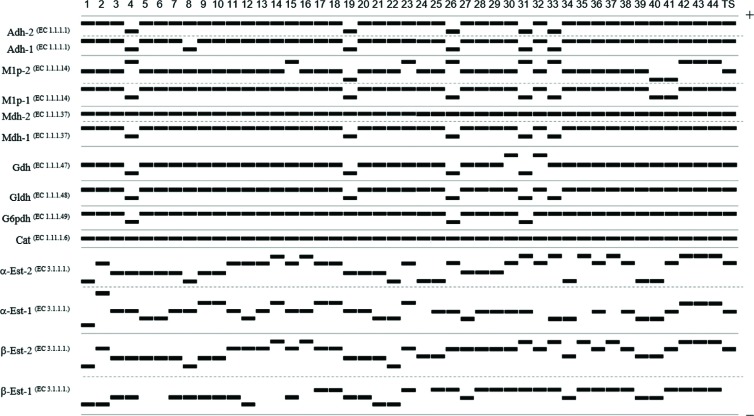
Diagram of the multilocus enzyme electrophoresis (MLEE) patterns of the *S. aureus* isolates. The migration of the enzymes occurred from the negative pole (cathode) to the positive pole (anode). The isolates are labeled from left to right 1 to 44 (TS corresponds to the *S. aureus* type strain – ATCC® 29213)

**Figure 3:**
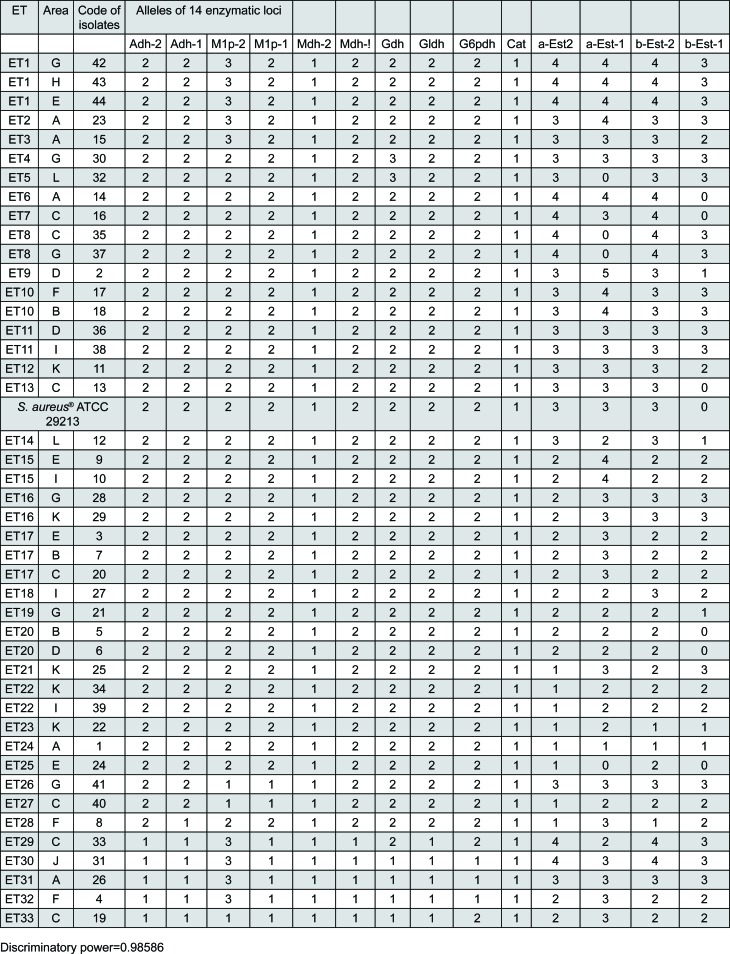
Allelic profiles of the 44 *S. aureus* isolates (33 S. aureus strains ETs) isolated from clinical environments (air) at the School of Dentistry at Piracicaba, State University of Campinas, São Paulo, Brazil

The *S. aureus* isoenzymatic patterns were reproducible in different gels after three repetitions of each electrophoretic run. Based on the haploid nature of *S. aureus*, such patterns indicate the following characteristics (Figures 2 and 3): 12 (85.7%) enzymatic loci were polymorphic for two, three, four, and five alleles (2 alleles: Adh-1, Adh-2, M1p-2, Mdh-1, Gldh, and G6pdh; 3 alleles: M1p-1, Gdh, β-Est-1; 4 alleles: α-Est-1 and α-Est-2; 5 alleles: β-Est-1). The mean number of alleles *per* locus and the mean number of alleles *per* polymorphic locus were equal to 2.57 and 2.83, respectively. The combination of existing alleles in 14 enzymatic loci revealed 33 electrophoretic types (ETs) (75% of the total isolates), that is, identical isolates corresponding to the same strain or ET (i.e., *d_ij_*=0.000). Only two loci were monomorphic for only one allele, Mdh-2 and Cat. The discriminatory power of the MLEE method, which is based on the numeric interpretation of electrophoretic patterns, was 0.98586, that is, a 98.6% probability that two unrelated *S. aureus* isolates sampled from the test population will be classified as different types (strains or ETs).

**Figure 4:**
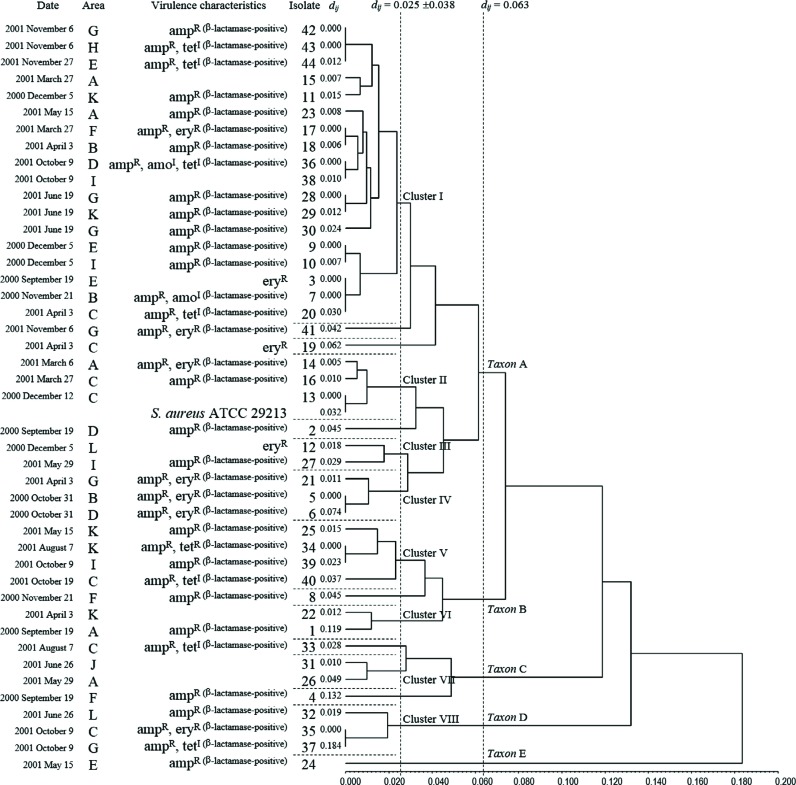
Genetic diversity of the 44 *S. aureus* isolates from the dental clinic environments (air) and the *S. aureus* type strain (ATCC® 29213). UPGMA dendrogram (*r_jk_*=0.83459) obtained from the genetic distance matrix *d_ij_*25. The letters A to L correspond to the sampling sites

The genetic diversity among the *S. aureus* isolates/strains was evaluated through the matrix *d_ij_* and a UPGMA dendrogram (Figure 4). When considering the threshold obtained (0≤*d_ij_*<0.025: highly related or identical isolates; 0.025≤*d_ij_*<0.063: moderately related isolates; 0.063≤*d_ij_*<0.184: distantly related isolates), the results showed five major groups or *taxa* (i.e., singular *taxon* – a taxonomic group of any nature or rank), named A, B, C, D, and E. The *taxon* A comprised three isolates (2^ET9 - area D^, 19^ET33 - area C^, 41 ^ET26 – area G^) and four moderately related clusters (from I to IV and totaling 29 isolates ^65.9%^ or 21 ETs ^60.6%^):

Cluster I: 18 highly related and/or identical isolates ^40.9%^ (isolates 3 ^ET17 – area E^, 7 ^ET17 – area B^, 9 ^ET15 – area E^, 10 ^ET15 – area I^, 11 ^ET12 – area K^, 15 ^ET3 – area A^, 17 ^ET10 – area F^, 18 ^ET10 – area B^, 20 ^ET17 – area C^, 23 ^ET2 – area A^, 28 ^ET16 – area G^, 29 ^ET16 – area K^, 30 ^ET4 – area G^, 36 ^ET11 – area D^, 38 ^ET11 – area I^, 42 ^ET1 – area G^, 43 ^ET1 - area H^, and 44 ^ET1 - area E^), or 10 highly related ETs ^30.3%^ obtained from 10 areas ^83.3%^ during ±14 months (from Sept. 19, 2000 to Nov. 27, 2001).

Cluster II: 3 highly related isolates ^6.8%^ (isolates 13 ^ET13 – area c^, 14 ^ET6 – area A^, and 16 ^ET7 - area C^), or 3 highly related ETs ^9.1%^, including *S. aureus* ATCC 29213, obtained from 2 ^16.7%^ areas during ±3 months (from Dec. 12, 2000 to March 27, 2001).

Cluster III: 2 highly related isolates ^4.5%^ (isolates 12 ^ET14 - area L^ and 27 ^ET18 - area I^), or 2 highly related ETs ^6.1%^ obtained from 2 ^16.7%^ areas during ±5 months (from Dec. 5, 2000 to May 29, 2001).

Cluster IV: 3 highly related isolates ^6.8%^ (isolates 5 ^ET20 - area B^, 6 ^ET20 - area D^, and 21 ^ET19 - area G^), or 2 highly related ETs ^6.1%^ obtained from 3 ^25%^ areas during ±6 months (from Oct. 31, 2000 to Apr. 3, 2001).

*Taxon* B comprised one isolate (isolate 8 ^ET28 - area F^) and two moderately related clusters (V and VI; totaling 7 isolates ^15.9%^ or 6 ETs ^18.2%^):

Cluster V: 4 highly related and/or identical isolates ^9.1%^ (isolates 25 ^ET21 - area K^, 34 ^ET22 - area K^, 39 ^ET22 - area I^, and 40 ^ET27 - area C^), or 3 highly related ETs ^9.1%^ obtained from 3 areas ^25%^ during ±5 months (from May 15, 2001 to Oct. 19, 2001).

Cluster VI: 2 highly related isolates ^4.5%^ (isolates 1 ^ET24 - area A^ and 22 ^ET23 - area K^), or 2 highly related ETs ^6%^ obtained from 2 areas ^16.7%^ during ±7 months (from Sept. 19, 2000 to Apr. 3, 2001).

*Taxon* C comprised two isolates (4 ^ET32 - area F^ and 33 ^ET29 - area C^) and one moderately related cluster (VII; totaling 4 isolates ^9.1%^ or 4 ETs ^12.1%^):

Cluster VII: 2 highly related isolates ^4.5%^ (31 ^ET30 - area J^ and 26 ^ET31 - area A^), or 2 highly related ETs ^6.1%^ obtained from 2 areas ^16.7%^ during ±1 month (from May 29, 2001 to June 26, 2001).

**Figure 5:**
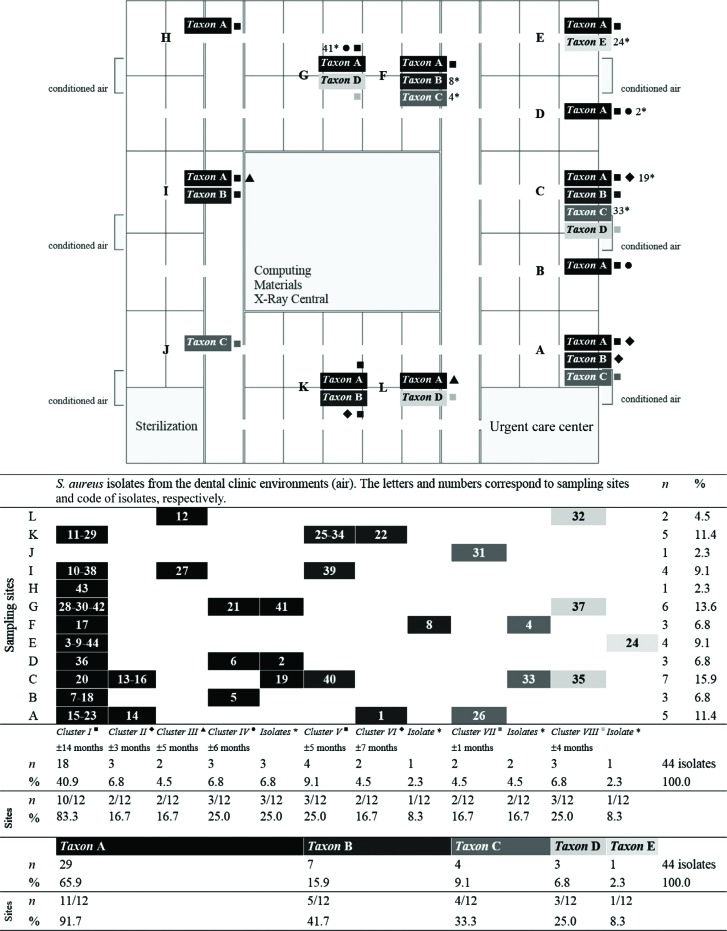
Design of the Academic Dental Clinic (School of Dentistry at Piracicaba, State University of Campinas, Brazil). The letters A to L are the sampling sites (Cluster I ■, Cluster II ♦, Cluster III ▲, Cluster IV •, Cluster V ■, Cluster VI ♦, Cluster VII ■, Cluster VIII ■ and isolates nonclustered*)

*Taxa* D and E comprised only one cluster [Cluster VIII: 3 highly related and/or identical isolates ^6.8%^ (32 ^ET5 - area L^, 35 ^ET8 - area C^, 37 ^ET8 - area G^), or 2 highly related ETs ^6.1%^ obtained from 3 areas ^25%^ during ±4 months (from June 26, 2001 to Oct. 9, 2001)] and one isolate (isolate 24 ^2.3%^ or ET25 ^3%^ obtained from area E), respectively. These results indicate that highly related or identical *S. aureus* isolates can emerge or remain for long periods in the same or different areas of dental office environments (Figures 4 and 5).

## Discussion

Strain delineation using multilocus enzyme electrophoresis (MLEE) allows for evaluations of genetic diversity and population structure analyses and provides a high discriminatory power and reproducibility^2^ ^,^ ^3^. Considered neutral markers (i.e., invariable when they suffer environmental selective pressures), metabolic isoenzymes have demonstrated a significant potential for the taxonomic, systematic, genetic, evolutionary, and epidemiological characterization of bacteria and yeasts of medical importance^2^ ^,^ ^3^ ^,^ ^23^ ^,^ ^26^ ^,^ ^28^.

In this study, the enzyme electrophoretic profiles of *S. aureus* samples from different gels were reproducible after three repetitions of each electrophoretic run. The high discriminatory capacity (i.e., >98% probability that two unrelated *S. aureus* isolates sampled from the test population will be classified as different types or strains) of MLEE, which is based on the interpretation of enzyme electrophoretic patterns, was also observed (i.e., the combination of existing alleles in 14 enzymatic loci revealed 33 ETs). MLEE has again been proven to be a powerful tool for typing *S. aureus* strains for epidemiological studies. The results related to the reproducibility and discriminatory power of MLEE corroborate with those previously reported by other medically relevant studies^24^ ^,^ ^28^; however, the discriminatory power described here is higher than those reported by other groups^7^ ^,^ ^17^.

Genetic polymorphisms have been reported in almost all natural populations, at all levels of genetic organization and from genotypic characteristics to phenotypic traits. The possible reasons for their existence have been the object of a long debate in population genetics and molecular evolution^10^ ^,^ ^11^. *S. aureus* is a heterogeneous (polymorphic) species^8^ that has a clonal population structure^6^. Therefore, it is believed that *S. aureus* does not undergo extensive recombination, diversifies largely by nucleotide mutations, and displays a high degree of linkage disequilibrium (nonrandom associations between genetic loci). A certain structural genetic locus is defined as polymorphic when the frequency of its most common allele presents a value below 0.99 (99%). Some of the measurements used to quantify this variability in organism populations are the gene and allele frequencies, the polymorphic loci percentage, the average number of alleles *per* locus, the average number of alleles *per* polymorphic locus, and the heterozygosity^14^. In this study, quantitative and qualitative variations of the polymorphic loci (12 ^85.7%^ enzymatic loci polymorphic for two, three, four, and five alleles), the average number of alleles *per* locus (2.57) and the average number of alleles *per* polymorphic locus (2.83) were observed in *S. aureus* population isolated from dental clinic environments (air). These variations have been observed in several genetic diversity studies of *S. aureus* populations recovered from human and bovine sources^7^ ^,^ ^8^ ^,^ ^17^ ^,^ ^22^. In addition, the genetic polymorphisms found among *S. aureus* isolates revealed a polyclonal pattern for air dispersion over time. However, monoclonal air dispersion was also observed for some of the isolates (isolates 9 ^ET15^ and 10 ^ET15^, 42^ET1^ and 43^ET1^, 36 ^ET11^ and 38 ^ET11^, 28 ^ET16^ and 29 ^ET16^, 5 ^ET20^ and 6 ^ET20^, 35 ^ET8^ and 37 ^ET8^) and for some of the periods of time, ranging from <1 to ±7 months (isolates 42 ^ET1^, 43 ^ET1^ and 44 ^ET1^ for <1 month, isolates 17 ^ET10^ and 18 ^ET10^ for <1 month, isolates 3 ^ET17^, 7 ^ET17^ and 20 ^ET17^ for ±7 months, and isolates 34 ^ET22^ and 39 ^ET22^ for ±2 months).

The genetic relationship among the *S. aureus* strains could be explained using Nei's distance *d_ij_* ^25^ and a UPGMA dendrogram^3^, according to the Pearson product-moment correlation coefficient value (*r_jk_*=0.83459) [i.e., good concordance between the *d_ij_* (genetic distance matrix) and *C_jk_* elements (correlation matrix derived from the UPGMA dendrogram)]. A high degree of genetic polymorphism (0.000≤*d_ij_*<0.184) was found among the clinical isolates (i.e., on average, from zero to 18.4 allelic substitutions were detected in every 100 loci from a common ancestral strain). Such isolates were distributed into five *taxa* (A to E), which established a genetic distance of 0.063≤*d_ij_*<0.184. *Taxon* A contained a larger number of isolates, strains or bacterial clusters (29 isolates ^65.9%^, 20 ETs ^60.6%^, and 4 clusters ^I,II,III and VI^), followed by *taxon* B (7 isolates ^15.9%^, 6 ETs ^18.2%^, and 2 clusters ^V and VI^), *taxon* C (4 isolates ^9.1%^, 4 ETs ^12.1%^, and 1 cluster ^VI^), *taxon* D (3 isolates ^6.8%^, 2 ETs ^6.1%^, and 1 cluster ^VIII^), and *taxon* E (1 isolate ^2.3%^ and 1 ET ^3%^). Each *taxon* contained one or more moderately related clusters and/or isolates (0.025≤*d_ij_*<0.063). In turn, these clusters harbored two or more highly related isolates (0≤*d_ij_*<0.025). These results reinforce the hypothesis that *S. aureus* is dispersed in clinical microenvironments (multi-user and urgency clinics and sterilization sectors) following both monoclonal and polyclonal patterns during a certain period and/or re-emerging temporarily. However, when considering that highly related strains come from a common ancestor [i.e., descendants have undergone microevolution and adaptations as a consequence of recombination (not extensive), nucleotide mutations, and nonrandom associations between genetic loci (linkage disequilibrium)^6^ ^,^ ^8^], these data also suggest the possibility of microevolutionary processes in *S. aureus* populations, as demonstrated in each cluster (i.e., on average, from zero to 2.5 allelic substitutions were detected in every 100 loci from a common ancestral strain) from ±1 to ±14 months. The existence of transmission methods and the horizontal/vertical spreading of such strains must be further explored.

Several sources might contribute to the appearance and reappearance of these strains in a clinical environment, which include such avenues as healthy carriers (patients, dental surgeons, and auxiliary staff) to the previous contamination of central air conditioning systems. As a consequence, microorganisms would be maintained in the air of dental environments for prolonged periods of time, which are characterized as spaces that might serve to spread microbial contamination. The high frequency of identical or highly related strains in the air of different environments might be due to the intense traffic of carriers such as students, assistants or professors. On the other hand, the relatively low frequency of *S. aureus* strains can be explained by the admission of new students/patients, which only reside in clinical environments for short periods of time. In addition, no correlation was observed between *S. aureus* strains (or clusters) and virulence characteristics, such as the production of β-lactamase and resistance to ampicillin, erythromycin, and tetracycline.

Certain aspects of practicing Dentistry may contribute to the transmission of microorganisms. Skin, environment, and instruments can be contaminated with saliva, blood or organic debris during routine dental treatment^29^. In the dental environment, investigators have observed an increase in the amount of microorganisms during clinical procedures, suggesting contamination by aerosols, especially when high-speed handpieces or ultrasonic scalers are used^1^ ^,^ ^20^. Among the species identified in microbiological studies, *Streptococcus viridans* and *Staphylococcus* spp. are the most prevalent microorganisms found on the surfaces of dental equipment^1^ ^,^ ^12^ ^,^ ^20^. In addition, the high-speed drills and cavitrons used in dental offices generate aerosols and droplets that are contaminated with blood and bacteria and may be a route for the transmission of diseases, such as SARS (severe acute respiratory syndrome), tuberculosis, and Legionnaires' disease^13^ ^,^ ^15^ ^,^ ^16^. Methicillin-resistant *S. aureus* (MRSA) has frequently been detected on surfaces in dental operatories, including the air-water syringe and reclining chair^18^. Nosocomial infections or the colonization of MRSA occurred in eight out of 140 patients who showed no evidence of MRSA upon admission to a clinic. Antibiogram tests revealed that the isolates from the eight patients were of the same strain as those from the surfaces of the dental operatory, suggesting *S. aureus* transmission between the patients and the dentist via the clinical environment^18^. Beyond their resistances to antimicrobial agents, *S. aureus* strains have the capacity to survive on dry surfaces for an average of 5 days^9^. Recently, the frequency of *S. aureus* isolated from the noses, hands, and tongues of students and patients and from the clinical environment of a pediatric Dentistry clinic before and after dental treatment was determined^24^. The highest concentration of *S. aureus* was found in the noses and on the tongues of children and among the dental students, and the highest level of contamination was observed on gloved hands, which was followed by the tongue and hands without gloves before clinical care. At the end of dental treatment, *S. aureus* colonies isolated from the gloved hands of students decreased significantly. Considering the clinical environment, *S. aureus* dissemination increased at the end of dental procedures, and the most contaminated areas were the auxiliary table and the storeroom, which was located at the center of the clinic. Such results can be explained by the intense circulation of people in the clinic and the use of high-speed dental handpieces. However, it is still speculated that much of the *S. aureus* contamination detected in the clinical environment came from other sources, such as direct contact, skin exfoliation or the improper handling of plates, and it is concluded that the dental clinic is an appropriate environment for *S. aureus* cross-transmission.

The survival of *S. aureus* on white coats over a 24-h period was investigated by van der Reijdena and colleagues^30^ (2009). Immediately after inoculation, only 0.16% of the cells survived. The number of viable cells decreased further to 0.046% after 1 h and to 0.014% after 24 h. To explore the capacity of bacteria to transfer from a white coat to another surface, the number of viable bacteria following a transfer to a stainless steel disc was also determined^30^. The number of recovered cells after contacting the stainless steel surface was not significantly different from the number before the contact, regardless of the dryness of the coat. Even airborne transfers of bacteria by moving contaminated pieces of white coat over agar plates in a disinfected laminar flow cabinet revealed a comparably low recovery of pathogenic bacteria after a direct recovery from the fabric^30^. The use of white coats in dental and medical clinics has been a common practice to prevent the contamination of healthcare workers by the pathogenic microorganisms found on patients or *vice versa.* It is well known that aerosols of oral secretions are frequently produced during dental interventions and that these secretions contain large numbers of microorganisms^27^.

The persistent presence of microorganisms on patient-derived dental impressions and gypsum casts and preliminary surveys of the practices and attitudes of 59 general dentists in Japan, concerning cross-infection control and their awareness of the possibility of microbial contamination on dental impressions and gypsum casts, were analyzed. The results demonstrated the presence of streptococci (100%), staphylococci (65.4%), *Candida* (46.2%), methicillin-resistance *S. aureus* (MRSA) (15.4%), and *Pseudomonas aeruginosa* (7.7%) on the impressions and the gypsum casts. Only 54% of the general dentists had a cross-infection policy in their dental clinics, and only 30% to 40% were aware of the possible persistence of MRSA or *P. aeruginosa* on impressions and gypsum casts. This knowledge confirmed the need for dental clinics to use appropriate infection control procedures and to prevent the possibility of cross-contamination, which results in infections by opportunistic pathogens, among patients and dental office and laboratory personnel^5^.

In a previous study, the contamination degree and antimicrobial susceptibility of *S. aureus* isolated from different touchable surfaces before, during, and after clinical procedures revealed an increase in the number of microorganisms during clinical procedures^19^. In addition, that study provided evidence that clinical procedures increased the number and proportion of antimicrobial-resistant *S. aureus* isolates dispersed in a dental clinic environment and highlighted the need to establish strategies to prevent the emergence of drug-resistant bacterial strains in dental settings. In this context, infection control guidelines and published research pertinent to dental infection control principles and practices, such as the one revised by the Centers for Disease Control and Prevention (CDC), must be applied by the dentist as a matter of routine in academic dental offices^16^.

## Conclusion

Because molecular-based epidemiological studies are useful in identifying possible sources of the spread of microorganisms in dental hospitals and clinical settings, this study contributes to our knowledge on the dynamics of the spread and seasonal retention of *S. aureus* strains resistant to antibiotics and points to the need for containment barriers, use of personal protective equipment, periodic identification and treatment of carriers among clinical staff, and installation of air purifiers. Finally, the MLEE method associated with genetic and clustering analysis allowed to identify a high degree of genetic polymorphism among *S. aureus* isolates, mono- and polyclonality patterns (bacterial strains dispersion in air environment of the academic dental clinic over time), *taxa* and clusters displaying variable frequencies of strains and possibly microevolutionary changes.
